# Psychological resilience and psychological status among community-dwelling older adults: a cross-sectional study in China

**DOI:** 10.3389/fpsyg.2026.1884231

**Published:** 2026-09-29

**Authors:** Xuanyu Chen, Jiaqi Zheng, Halen Maclaren, Xican Yin, Yinjia Zheng, Huihe Zhang

**Affiliations:** 1 College of Education, Psychology & Social Work Flinders University, Adelaide, SA, Australia; 2 Key Laboratory of Cognition and Personality (Southwest University), Ministry of Education, Chongqing, China; 3 School of Humanities, Jiangxi University of Chinese Medicine, Nanchang, China; 4 School of Humanities, Southeast University, Nanjing, China; 5 Teaching Management Section, The Fifth Affiliated Hospital of Guangzhou Medical University, Guangzhou, China; 6 Wenzhou Traditional Chinese Medicine Hospital of Zhejiang Chinese Medical University, Wenzhou, China

**Keywords:** culture, environment, old adults, psychological resilience, psychological status

## Abstract

**Introduction:**

In aging societies, promoting psychological well-being among older adults is crucial. While psychological resilience is known to benefit mental health, less is understood about how external resources—specifically cultural and environmental resilience—influence psychological status (life satisfaction and depression) via resilience pathways in older populations. This study investigated these associations and their cross-sectional indirect associations through psychological resilience.

**Methods:**

A cross-sectional survey was conducted with 508 community-dwelling adults aged ≥60 in Wenzhou, China (July–September 2024). Validated scales measured life satisfaction (SWLS), depression (CES-D-10), psychological resilience (BRS), cultural resilience (Chinese Cultural Beliefs of Adversity Scale), and environmental resilience (community walkability). Control variables included demographics, health, and financial strain. Data were analyzed using correlation, regression, and mediation analysis (PROCESS macro, Model 4) with bootstrapping (95% CI).

**Results:**

Psychological resilience partially mediated the relationships between both cultural resilience and environmental resilience with life satisfaction. Cultural resilience had a significant total effect on life satisfaction, with 28.9% of its effect mediated by psychological resilience. Environmental resilience’s total effect on life satisfaction was mediated by psychological resilience by 19.9%. However, psychological resilience did not mediate the relationships between either cultural or environmental resilience and depression.

**Conclusion:**

Psychological resilience serves as a significant mediator linking external resources (cultural and environmental resilience) to life satisfaction, but not depression, among older adults. Longitudinal research is needed to confirm causality.

## 1 Introduction

The psychological status of older adults has emerged as a critical concern in ageing societies (Song and Li, 2023). With declining fertility rates and rising life expectancy, China is undergoing profound population ageing; by 2050, individuals aged 65 and older are expected to comprise over 30% of the population (Luo et al., 2021). Against this demographic backdrop, promoting the psychological health of older adults is essential for achieving healthy ageing, a process of developing and maintaining functional ability that enables well-being in later life (World Health Organization, 2020).

In recent years, increasing attention has been directed toward the protective role of psychological resilience in older populations (Färber and Rosendahl, 2020; Trică et al., 2024; Zhang et al., 2025). Psychological resilience refers to the capacity to adapt positively and recover from stress, adversity, or major life events (Smith et al., 2008). Growing evidence indicates that psychological resilience is positively associated with healthy ageing (Jeste et al., 2013; Trică et al., 2024), life satisfaction (Smith and Hollinger-Smith, 2015), and reduced depressive symptoms (Wermelinger Ávila et al., 2017).

Given the established associations between psychological resilience and older adults’ mental health, scholars have increasingly examined the individual and contextual correlates of psychological resilience. Evidence suggests that the formation and development of psychological resilience are shaped by both intrinsic individual characteristics and external environmental resources (Greve and Staudinger, 2015). However, traditional resilience research has disproportionately emphasized internal psychological attributes while neglecting the contributions of external ecological systems such as the social, cultural, and built environment (Ungar, 2011). Addressing this gap, Ungar (2011) proposed the Social Ecology of Resilience framework, which posits that resilience emerges from dynamic interactions between individuals and their social and physical environments, that access to culturally appropriate environmental resources is crucial for fostering resilience, and that resilience involves dual mechanisms of navigation (accessing resources) and negotiation (constructing culturally meaningful interpretations of adversity).

Drawing on this social-ecological perspective, the present study focuses on two external resource systems: cultural beliefs about adversity and perceived community walkability, conceptualized here as specific facets of cultural resilience and environmental resilience, respectively. In this study, cultural resilience is operationalized as Chinese cultural beliefs about adversity, which is a culturally grounded meaning system through which older adults interpret hardship (Shek, 2005). Environmental resilience is operationalized as perceived community walkability for the ease of reaching 14 everyday destinations, which represents one accessibility dimension of place-based environmental resources (Sau Po Centre on Ageing, 2012). These indicators are not intended to exhaust the broader constructs; rather, they capture theoretically meaningful facets of cultural and environmental resilience that may be associated with psychological outcomes among older adults. These external resource systems may be directly associated with the psychological status of older adults and may also show indirect associations with life satisfaction and depressive symptoms through their associations with individual psychological resilience.

However, limited empirical research has systematically examined how cultural resilience and environmental resilience are jointly associated with psychological status through psychological resilience in older community-dwelling populations. Accordingly, the present study adopts a social-ecological perspective to investigate the interrelations among cultural resilience, environmental resilience, psychological resilience, and psychological status in older adults. Given the cross-sectional design, all analyses examine statistical associations rather than causal effects.

## 2 Literature review and research hypotheses

### 2.1 Relationship between psychological resilience and psychological status

Life satisfaction refers to an individual’s subjective cognitive evaluation of overall life quality (Pavot et al., 1991) and serves as a core indicator of psychological well-being in older adulthood (Bramhankar et al., 2023). According to the Assimilation–Accommodation Model, older adults recalibrate their aspirations and goals to maintain a sense of life quality when facing age-related challenges (Brandtstädter and Greve, 1994). Those with higher psychological resilience are more capable of drawing on internal strengths to cope with adversity, thereby maintaining or enhancing life satisfaction (Zhang, 2019). The positive association between psychological resilience and life satisfaction in later life has been well established (Zheng et al., 2020). Research on older migrants has found that greater resilience was significantly associated with higher life satisfaction (Akbar et al., 2014). Meta-analyses have further confirmed a significant positive association between resilience and psychological well-being indicators, including life satisfaction (Färber and Rosendahl, 2020; Hu et al., 2015). Older adults with higher psychological resilience are better positioned to cope with mental health concerns and physical health challenges (Liao et al., 2022).

In addition to life satisfaction, depression represents another critical aspect of psychological status in ageing populations (Bianchi et al., 2005). A robust body of evidence demonstrates that psychological resilience is negatively associated with depressive symptoms (Miller and Chandler, 2002). A meta-analysis by Wermelinger Ávila et al. (2017) confirmed this inverse relationship among older adults, and longitudinal research further suggests that sustained or increased resilience is associated with reduced mortality risk (Zhang et al., 2025).

### 2.2 Factors associated with psychological resilience

Psychological resilience in older adults is associated with environmental conditions and ecological contexts (Zheng et al., 2020). Staudinger et al. (1993) proposed a “person-in-context” framework, emphasizing that supportive, age-friendly environments play a vital role in fostering resilience. Similarly, Greve and Staudinger (2015) argued that resilience develops through continuous, dynamic interplay between the individual and their surrounding environmental systems.

A substantial body of evidence demonstrates that social support, community involvement, and neighborhood cohesion are positively associated with psychological resilience in older adults (Blane et al., 2011; Hildon et al., 2010; Wells, 2010). Building on this ecological understanding, Keck and Sakdapolrak (2013) advanced the concept of “environmental resilience” to describe the capacity of physical and social environments to enable individuals to maintain or enhance well-being and functioning under stress. Within the specific context of community-dwelling older adults, environmental resilience encompasses characteristics such as neighborhood safety, walkability, access to amenities and services, and supportive neighborhood relationships (Walsh, 2020; World Health Organization, 2007). In the present study, environmental resilience is operationalised specifically as perceived community walkability—the ease of reaching 14 everyday destinations—which represents one accessibility dimension of this broader construct rather than an exhaustive measure. These environmental attributes provide practical functional support that reduces environmental press and sustains autonomy in daily life (Nahemow and Lawton, 1973; Wahl et al., 2012). We therefore hypothesize that perceived community walkability is positively associated with psychological resilience among older adults.

In addition to environmental factors, cultural influences have recently gained increasing scholarly attention as critical resources for resilience (Kirmayer et al., 2011; Kramer et al., 2002). Culture provides the symbolic and moral frameworks through which adversity, ageing, and loss are interpreted, thereby shaping how stress is appraised and managed (Panter-Brick, 2014; Sen, 2004). From a social-ecological perspective, resilience is culturally embedded, as norms, values, and shared meanings determine which coping responses are recognized and supported as adaptive within a given context (Ungar, 2008, 2011). In Chinese cultural settings, traditional beliefs emphasizing endurance, balance (Zhongyong), acceptance, and moral self-cultivation—rooted in Confucian, Daoist, and Buddhist traditions—have been found to buffer helplessness and sustain motivation in the face of adversity (Cui et al., 2022; Shek, 2004). Research among Indigenous populations has similarly emphasized that cultural values, identity, and continuity serve as core drivers of psychological resilience (Kirmayer et al., 2011). These culturally grounded resources provide older adults with stable psychological support, meaning structures, and a sense of belonging, thereby reinforcing their capacity to adjust and recover from adversity. Of particular importance for the present study, we draw on the theoretical distinction between cultural resilience as a broader systemic capacity embedded in shared cultural resources (Ungar, 2013) and traditional cultural beliefs about adversity as an individual-level antecedent variable. Specifically, we conceptualize traditional cultural beliefs about adversity—positive identification with culturally rooted values regarding endurance, balance, and meaning in hardship—as an antecedent variable that contributes to psychological resilience, rather than as resilience itself. This conceptualization acknowledges that cultural beliefs serve as a cultural cornerstone for building psychological resilience (Kirmayer et al., 2011). In the present study, cultural resilience is operationalized specifically as traditional Chinese cultural beliefs about adversity, measured by the CCBA scale, which captures a specific facet of the broader cultural resilience construct. Based on this theoretical reasoning, we hypothesize that traditional cultural beliefs about adversity are positively associated with psychological resilience.

In summary, both perceived community walkability (as an indicator of environmental resilience) and traditional cultural beliefs about adversity (as an indicator of cultural resilience) not only may show direct associations with the psychological well-being of older adults but may also be indirectly associated with psychological resilience.

### 2.3 Potential mediating effects of psychological resilience

Extensive research has examined psychological resilience as a potential statistical mediator that links ecological resources to mental health outcomes in older populations. For instance, Liao et al. (2022) demonstrated that resilience mediates the association between social engagement and life satisfaction among Chinese older adults. Studies involving older migrants further support this pattern: Kong et al. (2021) found that resilience mediates the impact of social support on health-related quality of life, while Wang et al. (2022) observed its mediating role in the relationship between loneliness and depressive symptoms. Beyond social contexts, Xin and Ma (2023) reported that resilience mediates the effect of physical exercise on anxiety reduction. Critically, diminished resilience resulting from social isolation has been found to correlate with decreased life satisfaction and elevated depressive symptoms (Zhao et al., 2018). Drawing on the preceding literature review and social-ecological theory, the present study hypothesizes that psychological resilience may serve as a statistical mediator through which both perceived community walkability and traditional cultural beliefs about adversity are associated with the psychological status—specifically, life satisfaction and depression—of community-dwelling older adults.

### 2.4 Research hypotheses

Based on the above discussion and earlier literature review, the research question is: How are perceived community walkability (as an indicator of environmental resilience) and cultural beliefs about adversity (as an indicator of cultural resilience) associated with the psychological status of older adults, and to what extent are these associations statistically mediated by psychological resilience? The following are the research hypotheses. All hypotheses are framed in associational terms because the study design is cross-sectional and cannot establish causal direction.

Hypothesis 1: Cultural beliefs about adversity (as an indicator relevant to cultural resilience) are associated with psychological status among older adults.

Hypothesis 2: The associations between cultural beliefs about adversity and psychological status are statistically mediated by psychological resilience.

Hypothesis 3: Perceived community walkability is associated with psychological status among older adults.

Hypothesis 4: The associations between perceived community walkability and psychological status are statistically mediated by psychological resilience.

Hypothesis 5: Psychological resilience is associated with psychological status among older adults.

## 3 Materials and methods

### 3.1 Participants

The participants were older adults aged 60 and above residing in Wenzhou, Zhejiang, China, where this age group accounted for 23.03% of the total population in 2024 (Qianjiang Evening News, 2024). Due to limited funding, a convenience sampling approach was adopted. The researcher identified common gathering places of older adults, such as local parks and community centers, and invited a total of 600 individuals to participate. Of these, 508 provided fully completed questionnaires, yielding a response rate of 84.7%. Ethical approval was obtained from the Flinders University Human Research Ethics Committee (Project No. 7285), after which data collection commenced. Informed written consent was obtained in accordance with local cultural practices, and participants were assured of confidentiality, anonymity, and their right to withdraw at any time.

### 3.2 Procedure

Data were collected from July to September 2024 through structured face-to-face interviews using a questionnaire consisting mainly of close-ended questions. This approach was chosen to ensure clarity and accessibility, given potential hearing or vision impairments among older adults. To encourage honest responses, anonymity and confidentiality were emphasized. Trained research assistants fluent in both Mandarin and the Wenzhou dialect conducted all interviews. All instruments were administered in their standard Chinese versions; when participants had difficulty understanding Mandarin or written items, research assistants provided explanations in the Wenzhou dialect while preserving the original item meaning. This procedure was designed to maintain cultural appropriateness and comprehension without altering the instruments’ content. Because the interviews were interviewer-administered and carefully monitored for completeness, no item-level missing data occurred; thus, no imputation procedures were applied.

### 3.3 Measures

#### 3.3.1 Psychological status

Psychological status was operationalized through life satisfaction and depression. Life satisfaction was assessed using the Satisfaction with Life Scale (SWLS; Diener et al., 1985), which comprises 5 items rated on a 6-point Likert scale (1 = strongly disagree, 7 = strongly agree). Total scores range from 5 to 30, with higher scores indicating greater life satisfaction. The Chinese version translated by Shek (1998) was used; it has demonstrated internal consistency ranging from 0.71 to 0.93 in Chinese samples (Bai et al., 2021; Zheng et al., 2020). In the present study, Cronbach’s alpha was 0.92.

Depression was measured using the 10-item Center for Epidemiologic Studies Depression Scale [CES-D 10; Chinese version translated by Wong (2009)]. Each item is scored from 0 (rarely or none of the time) to 3 (most or all of the time), yielding a total score ranging from 0 to 30, with higher scores reflecting greater depressive symptomatology. Items 4, 5, and 8 assess positive affect; the remaining items assess depressive symptoms. The 10-item version is a valid alternative to the 20-item CES-D (Cheng and Chan, 2006) and has shown internal consistency ranging from 0.82 to 0.86 in Chinese older adult samples (Huang et al., 2020; Huang et al., 2014). For the current study, Cronbach’s alpha was 0.79.

#### 3.3.2 Psychological resilience

Psychological resilience was measured using the 6-item Brief Resilience Scale (BRS; Smith et al., 2008). Each item is rated on a 5-point Likert scale (1 = strongly disagree, 5 = strongly agree). The BRS assesses the ability to bounce back from stress and adversity. Positively worded items (1, 3, 5) and negatively worded items (2, 4, 6) are combined after reverse coding the negative items, with higher scores indicating greater resilience. The Chinese version adapted by Huang et al. (2020) was employed and demonstrated high internal consistency in this study (Cronbach’s *α* = 0.91). The BRS has shown acceptable construct validity and measurement invariance across Chinese adult samples.

#### 3.3.3 Cultural resilience

Cultural resilience was measured using the Chinese Cultural Beliefs about Adversity (CCBA) Scale (Shek, 2005). The CCBA scale captures a specific facet of cultural resilience—namely, traditional cultural beliefs about adversity—rather than the full construct of cultural resilience. The scale contains 9 items, each rated on a 6-point Likert scale (1 = strongly disagree, 6 = strongly agree). Items 2 (“Life depends on fate”) and 5 (“Poverty stifles ambition”) assess negative beliefs; the remaining items assess positive beliefs. After reverse-coding items 2 and 5, all item scores are summed to produce a total score ranging from 9 to 54. Higher total scores indicate stronger endorsement of positive traditional cultural beliefs about adversity. The scale demonstrated good reliability in previous research (Shek, 2005), and Cronbach’s alpha was 0.83 in the current study. The CCBA has been used widely in Chinese contexts and has shown adequate construct validity for measuring culturally specific beliefs about hardship.

#### 3.3.4 Environmental resilience

Environmental resilience was operationalized as perceived community walkability. Perceived community walkability captures one accessibility dimension of environmental resilience—the ease of reaching everyday destinations—rather than the full construct. Respondents rated the ease of walking to 14 common community destinations on a 5-point Likert scale (1 = very difficult, 5 = very easy). The 14 destinations were drawn from research on community walkability in Hong Kong (Sau Po Centre on Ageing, 2012) and included transport stations, parks, community centers, shops, banks, hospitals, and friends’ homes. Item scores are summed to yield a total score ranging from 14 to 70, with higher scores indicating greater perceived walkability. The scale demonstrated high internal consistency (Cronbach’s *α* = 0.93). The walkability items were adapted from the Hong Kong community walkability assessment and were judged by local grassroots community workers to be culturally appropriate for Wenzhou neighborhoods.

#### 3.3.5 Control variables

Control variables included gender, age (in years), education (in years of formal schooling), marital status, self-rated health, and financial status. Self-rated health was measured with a single item: “How would you rate your present health?” Responses were recorded on a 5-point scale (1 = very poor, 2 = poor, 3 = fair, 4 = good, 5 = very good). Financial status was assessed using the 4-item Financial Strain Scale developed by Chou and Chi (2002). Each item evaluates whether participants have enough money to meet daily needs (food, medical services, daily expenses, monthly bills) using a 3-point scale (1 = enough, 2 = barely enough, 3 = not enough). Total scores range from 4 to 12, with higher scores indicating greater financial strain. In the present study, the scale showed high internal consistency (Cronbach’s *α* = 0.92). The Financial Strain Scale has been validated in Chinese older adult populations and demonstrates good construct validity.

### 3.4 Data analyses

Data were coded and analyzed using SPSS 28.0 for Windows. Frequencies and histograms were generated to examine variable distributions and identify outliers. Bivariate analyses were conducted to assess associations between independent variables and psychological status. Multivariate analyses, including correlation and multiple regression, were used to explore key relationships. The SPSS macro PROCESS (v4.2) was employed to test indirect effects with bias-corrected bootstrap confidence intervals (5,000 resamples).

## 4 Result

### 4.1 Descriptive statistics

Of the 508 participants, 47.4% were male and 52.6% were female (see Table 1). A total of 46.7% held an agricultural hukou, while 53.3% held a non-agricultural hukou. Regarding self-rated health, 1.0% reported very poor health, 5.1% poor, 33.1% fair, 43.9% good, and 16.9% very good. In terms of family harmony, 3.3% described their family relationships as very disharmonious, 4.1% as disharmonious, 17.7% as neutral, 51.2% as harmonious, and 23.6% as very harmonious. As shown in Table 1, participants’ ages ranged from 60 to 100 (M = 71.53, SD = 7.89). Years of formal education ranged from 0 to 32 (M = 6.33, SD = 4.17). One participant reported 32 years of formal education; this value was verified against the original interview record. Financial strain scores ranged from 4 to 12 (M = 6.62, SD = 2.55). Life satisfaction scores ranged from 5 to 30 (M = 22.44, SD = 5.50), and depression scores ranged from 0 to 23 (M = 8.98, SD = 6.53).

**Table 1:** Descriptive statistics of the variables (*N* = 508).

Variable	% or Mean (SD)	Range
Male (versus female)	47.4 (52.6)	–
Agricultural hukou (versus non-agricultural hukou)	46.7 (53.3)	–
Married (versus others)	82.3 (17.7)	
Self-perceived health		
Very poor	1.0	1–5
Poor	5.1	
Neutral	33.1	
Good	43.9	
Very good	16.9	
Age	71.53 (7.89)	60–100
Years of formal education	6.33 (4.17)	0–32
Financial strain	6.62 (2.55)	4–12
Life satisfaction	22.44 (5.50)	5–30
Depression	8.98 (6.53)	0–23
Psychological resilience	21.87 (5.18)	6–30
Cultural Resilience	41.32 (7.28)	14–54
Environmental Resilience	59.18 (10.28)	14–70

SD, standard deviation.

### 4.2 Predicting life satisfaction and depression

This study examined how cultural resilience and environmental resilience are associated with psychological status among older adults, and whether these relationships are carried by psychological resilience. Figure 1 illustrates the hypothesized mediation model and the covariations among the study variables.

**Figure 1:**
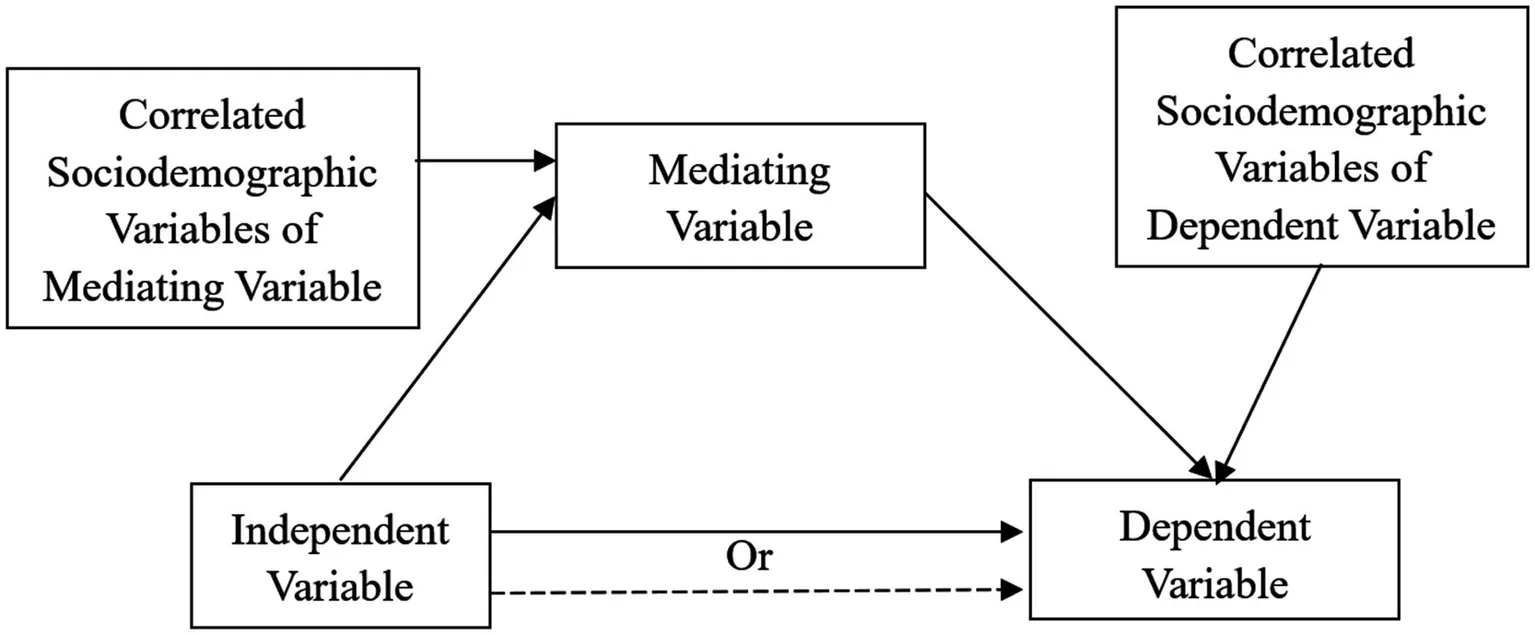
Relationship path of mediating model and covariations in variables.

### 4.3 Correlation and regression analyses

Regression analyses were conducted to examine the relationships between key variables and to test the research hypotheses. Prior to this, bivariate correlations were computed. Table 2 presents the Pearson correlation matrix. Notably, gender, age, marital status, and financial status were not significantly associated with life satisfaction, while gender, age, marital status, education, hukou status, and social network were not significantly associated with depression.

**Table 2:** The matrix of correlation coefficients for the variables.

Variables	1	2	3	4	5	6	7	8	9	10	11	12
1. G	–											
2. Age	−0.066	–										
3. E	−0.144**	−0.124**	–									
4. M	0.183**	0.257**	−0.133**	–								
5. HK	0.131**	−0.003	0.449**	0.072	–							
6. HS	0.076	−0.111*	0.097*	−0.047	0.007	–						
7. FS.	−0.025	0.091*	−0.141**	−0.065	−0.213**	−0.235**	–					
8. PR	0.021	−0.019	−0.093	−0.021	−0.065	0.204**	0.011	–				
9. CuR	0.176**	0.020	−0.068	0.004	−0.103*	0.119**	0.030	0.272**	–			
10. ER	0.031	0.010	−0.040	−0.012	−0.066	0.229**	−0.202**	0.312**	0.259**	–		
11. LS	0.024	0.098	−0.147**	−0.011	−0.225**	0.222**	−0.002	0.429**	0.325**	0.449**	–	
12. D	−0.023	0.050	−0.059	0.007	−0.011	−0.160**	0.326**	−0.150**	−0.230**	−0.356**	−0.237**	–

G, gender (female vs male); E, education; M, marital status (married vs others); HK, hukou status (agricultural hukou vs non-agricultural hukou); HS, health status; FS, financial strain; PR, psychological resilience; CuR, cultural resilience; ER, environmental resilience; LS, life satisfaction; D, depression; *p < 0.05; **p < 0.01; ***p < 0.001 (two-tailed test).

### 4.4 Mediating role of psychological resilience

Correlation analyses showed that cultural resilience, environmental resilience, and psychological resilience were significantly interrelated and were each associated with both life satisfaction and depression. These patterns justified formal testing of indirect associations. As a preliminary descriptive step, we conducted a series of regressions following the logic of Baron and Kenny (1986). Both cultural resilience and environmental resilience were significantly associated with life satisfaction and depression (Step 1), with psychological resilience (Step 2), and psychological resilience was significantly associated with life satisfaction after controlling for the two independent variables (Step 3). In Step 4, the regression coefficients of cultural resilience and environmental resilience on life satisfaction were attenuated when psychological resilience was added, suggesting that an indirect association might exist (see Table 3).

**Table 3:** Mediational analyses of life satisfaction.

Dependent variables	Step 1		Step 2		Step 3 and 4	
	Life satisfaction		Psychological resilience		Life satisfaction	
Indicator	*B* (SE)	*β* (Sig.)	*B* (SE)	*β* (Sig.)	*B* (SE)	*β* (Sig.)
Education	−0.082(0.056)	−0.062	–	–	−0.052 (0.054)	−0.039
Hukou status	−1.692(0.463)	−0.154***	–	–	−1.714 (0.444)	−0.156***
Health status	0.811(0.254)	0.124**	0.784 (0.260)	0.127**	0.574 (0.246)	0.088*
Cultural resilience	0.150(0.029)	0.198***	0.140 (0.030)	197***	0.111 (0.029)	0.148***
Environmental resilience	0.191(0.021)	0.357***	0.117 (0.022)	232***	0.159 (0.021)	0.297***
Psychological resilience	–	–	–	–	0.280 (0.042)	0.264***
*R* ^2^	0.296		0.152		0.355	
Adjusted *R*^2^	0.289		0.147		0.347	
*F* (Sig.)	42.245***		30.150***		45.906***	
Durbin–Watson	1.592		1.491		1.633	
Range of tolerance	0.788–0.931		0.893–0.944		0.782–0.912	
Range of VIF	1.074–1.270		1.060–1.119		1.097–1.279	

Hukou status (agricultural hukou vs non-agricultural hukou). Sig., significance. *p < 0.05; **p < 0.01; ***p < 0.001 (two-tailed test).

The core mediation evidence for life satisfaction was obtained through bootstrap analysis. We used the PROCESS macro for SPSS (Model 4; Hayes, 2013) with 5,000 bias-corrected bootstrap resamples. Two separate models were tested to isolate the indirect effect of each ecological resource. In the first model, cultural resilience was entered as the independent variable (X), psychological resilience as the mediator (M), and life satisfaction as the dependent variable (Y); education, hukou status, and health status were included as covariates. Environmental resilience was not included in this model. In the second model, environmental resilience served as X, with the identical mediator, outcome, and covariates, excluding cultural resilience.

The first model (Table 4) showed that the 95% bootstrap confidence interval for the indirect effect of cultural resilience on life satisfaction through psychological resilience did not include zero [indirect effect = 0.0612, 95% CI (0.0360, 0.0934)]. The total effect was 0.212, the direct effect was 0.1503, and the proportion mediated was 28.9%. Thus, psychological resilience partially carried the association between cultural resilience and life satisfaction.

**Table 4:** Direct and indirect effects of cultural resilience on life satisfaction.

Variables	Effect size (*B*)	SE/BootSE	95% CI	Proportion of effect
Path a: X → M	0.174	0.030	[0.114, 0.233]	—
Path b: M → Y	0.352	0.043	[0.2684, 0.436]	—
Total effect	0.212	0.031	[0.151, 0.272]	100%
Direct effect	0.150	0.030	[0.092, 0.209]	71.1%
Indirect effect	0.061	0.015	[0.035, 0.093]	28.9%

SE, standard error; CI, 95% confidence interval.

The second model (Table 5) similarly demonstrated that the indirect effect of environmental resilience on life satisfaction via psychological resilience was significant [indirect effect = 0.0430, 95% CI (0.0259, 0.0621)]. The total effect was 0.2161, the direct effect was 0.1731, and the proportion mediated was 19.9%. Psychological resilience therefore also partially carried the association between environmental resilience and life satisfaction. Separate mediation analyses indicated that psychological resilience statistically mediated the associations of both cultural resilience and environmental resilience with life satisfaction (see Figure 2).

**Table 5:** Direct and indirect effects of environmental resilience on life satisfaction.

Variables	Effect size (*B*)	SE/BootSE	95% CI	Proportion of effect
Path a: X → M	0.138	0.022	[0.096, 0.181]	—
Path b: M → Y	0.312	0.041	[0.231, 0.393]	—
Total effect	0.216	0.021	[0.175, 0.258]	100%
Direct effect	0.173	0.021	[0.133, 0.214]	80.1%
Indirect effect	0.043	0.009	[0.026, 0.062]	19.9%

**Figure 2:**
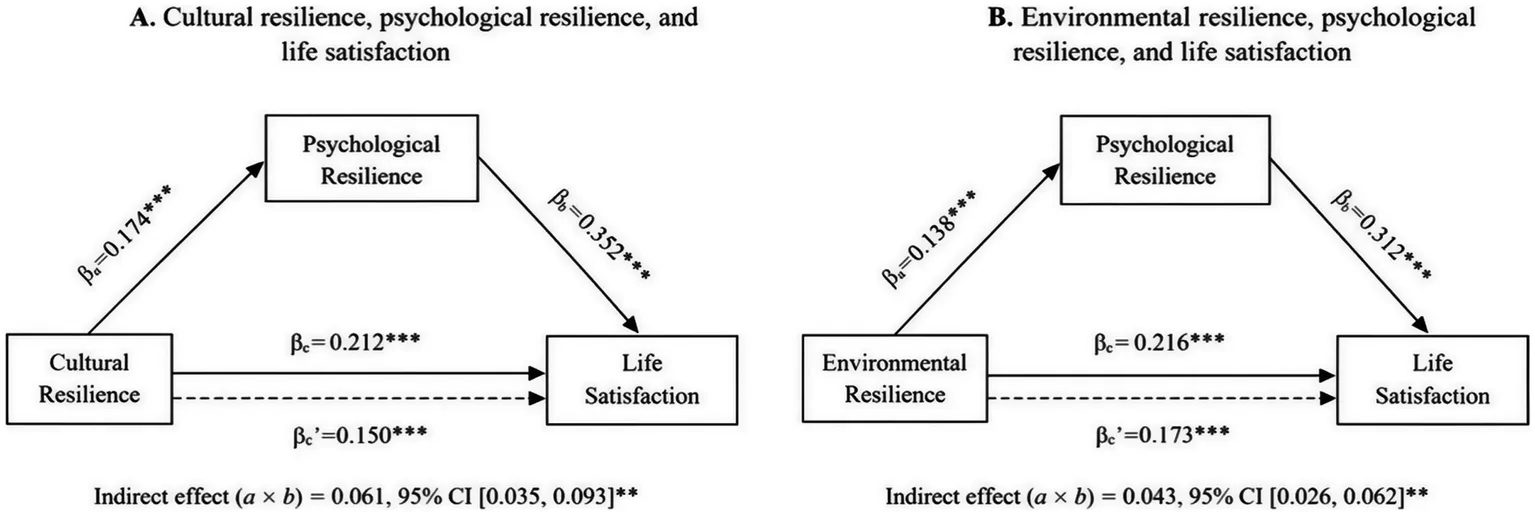
The path of psychological resilience in the relationship between cultural resilience, environmental resilience and life satisfaction. Values are unstandardized coefficients. ***p < 0.001. In the mediation model diagram, β denotes the standardized path coefficient (standardized regression weight). It represents the expected change in the outcome, in standard deviation units, associated with a one-standard-deviation increase in the predictor, holding the other predictors in the model constant. The sign of β indicates the direction of the effect, and its absolute value indicates the strength of the association.

All regression diagnostics were within acceptable limits. Durbin–Watson statistics ranged from 1.491 to 1.633, VIF values were below 2.0, tolerance statistics exceeded 0.2, and residual plots supported normality and homoscedasticity.

For depression (see Table 6), because psychological resilience was not significantly associated with the outcome after controlling for cultural and environmental resilience (Step 3, *β* = −0.029, *p* > 0.05), the basic precondition for mediation was not met. The addition of psychological resilience in Step 4 left the coefficients of cultural resilience (*β* changed from −0.171 to −0.165) and environmental resilience (*β* from −0.253 to −0.245) essentially unchanged. Bootstrap indirect effect confidence intervals (based on the same PROCESS specification) also contained zero, confirming the absence of mediation (see Tables 7, 8).

**Table 6:** Mediational analyses of depression.

Dependent variables	Step 1		Step 2		Step 3 and 4	
	Depression		Psychological resilience		Depression	
Indicator	*B* (SE)	*β*	*B* (SE)	*β*	*B* (SE)	*β*
Health status	−0.132 (0.321)	−0.017	0.784(0.260)	0.127**	−0.100 (0.325)	−0.013
Financial strain	0.706 (0.105)	0.276***	–	–	0.712 (0.106)	0.278***
Cultural resilience	−0.153 (0.037)	−0.171***	140 (0.030)	0.197***	−0.148 (0.038)	−0.165***
Environmental resilience	−0.160 (0.027)	−0.253***	117 (0.022)	0.232***	−0.156 (0.028)	−0.245***
Psychological resilience	–	–	–	–	−0.036 (0.054)	−0.029
*R* ^2^	0.222		0.152		0.223	
Adjusted *R*^2^	0.216		0.147		0.215	
*F* (Sig.)	35.865***		30.150***		28.749***	
Durbin–Watson	1.338		1.491		1.342	
Range of Tolerance	0.866–0.920		0.893–0.944		0.814–0.904	
Range of VIF	1.087–1.155		1.060–1.119		1.106–1.229	

PR, psychological resilience; CuR, cultural resilience; ER, environmental resilience; D, depression; Significance levels: *p < 0.05; **p < 0.01; ***p < 0.001 (two-tailed test).

**Table 7:** Direct and indirect effects of cultural resilience on depression.

Variables	Effect size (*B*)	SE/BootSE	95% CI
Path a: X → M	0.177	0.030	[0.118 0.237]
Path b: M → Y	−0.110	0.054	[−0.216, −0.004]
Total effect	−0.209	0.037	[−0.281, −0.136]
Direct effect	−0.189	0.038	[−0.264, −0.115]
Indirect effect	−0.020	0.010	[−0.04, 0.0002]

**Table 8:** Direct and indirect effects of environmental resilience on depression.

Variables	Effect size (*B*)	SE/BootSE	95% CI
Path a: X → M	0.150	0.022	[0.107, 0.193]
Path b: M → Y	−0.077	0.054	[−0.183, 0.029]
Total effect	−0.189	0.026	[−0.240, −0.137]
Direct effect	−0.177	0.028	[−0.231, −0.123]
Indirect effect	−0.012	0.008	[−0.029, 0.004]

In summary, psychological resilience partially mediated the relationships of both cultural resilience and environmental resilience with life satisfaction, but did not mediate their relationships with depression.

## 5 Discussion

Grounded in the social ecology of resilience framework (Ungar, 2011), this study examined the associations among traditional Chinese cultural beliefs about adversity (as an indicator of cultural resilience), perceived community walkability (as an indicator of environmental resilience), psychological resilience, and the psychological status of community-dwelling older adults in Wenzhou, China. Psychological status comprised both life satisfaction and depression. Consistent with the framework’s core premise—that resilience emerges from dynamic interactions between individuals and their social and physical environments—our findings indicate that cultural and environmental resources are significantly associated with life satisfaction, and that psychological resilience partially accounts for these associations.

The direct positive association between cultural resilience—operationalized through traditional Chinese cultural beliefs about adversity—and life satisfaction is consistent with prior research conducted among Chinese populations (Huang and Wu, 2012; Shek, 2005). Such cultural beliefs, which emphasize endurance, fate acceptance, and self-cultivation, have historically served as fundamental motivational resources for Chinese individuals striving toward satisfactory lives (Meili and Maercker, 2019; Shek, 2004). From a social-ecological perspective, these beliefs may function as culturally embedded meaning systems that are associated with how adversity is interpreted and managed, and might thus be related to psychological well-being. Older adults who more strongly endorse positive cultural beliefs about adversity may actively engage in efforts to overcome difficulties, perceive their life circumstances more favorably, and consequently report higher life satisfaction (Huang and Wu, 2012).

Importantly, our findings revealed that psychological resilience statistically mediated the association between cultural resilience operationalized through traditional Chinese cultural beliefs about adversity and life satisfaction, with the indirect pathway accounting for approximately 28.9% of the total effect. This mediation pattern aligned with the social-ecological view that environmental and cultural resources might be associated with individual-level capacities for positive adaptation. Several possible explanations might underlie this statistical mediation. First, cultural beliefs about adversity may be related to foundational schemas that support the development of psychological resilience (Souri and Hasanirad, 2011). By providing coherent frameworks for understanding hardship, these beliefs may be associated with greater cognitive flexibility and the capacity to reframe challenges in meaningful ways—constructs often considered core components of psychological resilience. Second, positive cultural beliefs about adversity share conceptual similarities with dispositional optimism (Huang et al., 2014), a well-established correlate of resilience (Tusaie-Mumford, 2001). Third, it is plausible that these cultural beliefs are indirectly associated with psychological resilience through enhanced self-esteem and sense of mastery, as prior research has demonstrated associations among these constructs (Aghaei et al., 2024; Shek et al., 2003), though we note that these specific pathways were not directly tested in the present study. Taken together, these interpretations suggest that traditional cultural beliefs about adversity, as one facet of cultural resilience, may be related to psychological resilience at the individual level, which in turn is associated with life satisfaction.

The direct association between environmental resilience operationalized as perceived community walkability and life satisfaction corroborates earlier work on walkable environments and well-being (Frank et al., 2010; Su et al., 2017; Zuniga-Teran et al., 2017) and extends this evidence to community-dwelling older adults in mainland China. Walkable community environments may be associated with life satisfaction through multiple pathways: they may support independent living by reducing environmental press, may facilitate social interaction and may strengthen social networks, and may promote physical activity that benefits both physical and cognitive health (Miller et al., 2000; Weuve et al., 2004). Social connectedness arising from such interactions may, in turn, be associated with a sense of self-worth and belonging, which may further be related to well-being (Antonucci et al., 2007).

Psychological resilience also statistically mediated the association between environmental resilience operationalized as perceived community walkability and life satisfaction, accounting for approximately 19.9% of the total effect. This finding suggests that the associations between walkable environments and life satisfaction may be partly indirect through strengthened individual psychological resilience. Consistent with the social-ecological framework’s emphasis on “navigation” to resources, perceived community walkability may be associated with older adults’ capacity to access and utilize environmental resources, and may therefore be related to a sense of mastery and self-efficacy. While it is plausible that increased self-efficacy is associated with psychological resilience (Benight and Cieslak, 2011), this pathway remains speculative as self-efficacy was not measured in the current study. Alternatively, walkable environments may be associated with social support and neighborhood cohesion, which are well-established correlates of psychological resilience (Giles-Corti et al., 2013; Koohsari et al., 2015; Ma et al., 2023; Yan et al., 2022). Through these interconnected associations, perceived community walkability, as one facet of environmental resilience, may be related to individual psychological resilience.

Contrary to our hypotheses, psychological resilience was not significantly associated with depression in the full regression model, nor did it statistically mediate the relationships of traditional cultural beliefs about adversity and perceived community walkability with depression. This null finding diverges from a substantial body of prior literature documenting inverse associations between resilience and depressive symptoms (Wermelinger Ávila et al., 2017). Several explanations merit consideration. First, the temporal frames of measurement differ: psychological resilience was assessed as a relatively stable capacity, whereas the CES-D 10 captures depressive mood over the preceding two weeks, a more transient and state-sensitive window. In contrast, life satisfaction reflects a more enduring cognitive evaluation (Diener, 2000; Pavot and Diener, 1993), which may align more closely with dispositional resilience. Second, the present sample was characterized by predominantly good self-rated health and relatively low depressive symptom levels due to the community-based convenience sampling strategy. This restricted variance in depression may have attenuated statistical associations with psychological resilience. Third, in a context where cultural and environmental resources are robust, these systemic protective factors may show stronger direct associations with depression than individual-level psychological resilience, effectively absorbing variance that would otherwise be attributed to resilience. This interpretation aligns with the social-ecological principle that resilience is distributed across multiple systems rather than residing solely within the individual (Ungar, 2011). We recommend that future research employing longitudinal designs and clinically diverse samples investigate whether the resilience–depression link is moderated by ecological resource availability.

The positive association between psychological resilience and life satisfaction is consistent with earlier studies (Netuveli and Blane, 2008; Zheng et al., 2020) and provides additional empirical support for theoretical frameworks that have proposed resilience as a protective factor, including the theory of psychological stress (Lazarus and Folkman, 1984), existentialist perspectives (Sixsmith, 1993), and the model of assimilation and accommodation (Brandtstädter and Greve, 1994).

With respect to control variables, better self-rated health was positively associated with life satisfaction, which may be explained by the possibility that healthier older adults have greater capacity to engage in personal hobbies, family roles, and social and recreational activities that are associated with life satisfaction (Pinquart and Sörensen, 2000). Non-agricultural hukou status was also associated with higher life satisfaction, consistent with the well-documented welfare disparities in China’s household registration system, where urban residents enjoy substantially higher pensions and social benefits (Wu and Treiman, 2004). Financial strain was positively associated with depression, a finding consistent with the broader literature linking economic hardship to psychological distress in later life (Chou and Chi, 2002; Krause et al., 1991).

### 5.1 Limitations

Several limitations warrant caution. First, convenience sampling at parks and community centers likely excluded homebound, frail, or severely depressed older adults. And only 1.0% rated their health as “very poor” and 5.1% as “poor” so the sample overrepresents healthier, more mobile individuals. This may inflate associations involving perceived walkability and limit generalizability to active older adults. Second, the cross-sectional design precludes causal inference; the mediation results represent statistical indirect associations and remain vulnerable to reverse causality. Third, all measures were self-reported, raising concerns about social desirability and common-method bias, while unmeasured confounders (e.g., personality, cognition, residential selection) were not controlled. Proposed intervening variables such as self-efficacy and mastery were also not directly assessed. Fourth, the operationalization captured only narrow facets of the broader constructs: cultural resilience was measured solely through traditional beliefs about adversity, and environmental resilience solely through perceived walkability, omitting features like safety, green space, and social cohesion. Finally, the sample was drawn exclusively from Wenzhou, which is an economically developed eastern Chinese city with a distinctive clan culture restricting generalizability to other regions and cultural settings. Future studies should combine longitudinal and multi-region designs with probability-based sampling and multi-method assessments, including objective indicators of the built environment where feasible.

### 5.2 Implications

Given the cross-sectional, observational design, these findings should be interpreted cautiously. Accordingly, implications are framed as hypotheses rather than causal conclusions. Theoretically, the results align with a social-ecological resilience perspective, suggesting that cultural beliefs about adversity and perceived neighborhood walkability may relate to individual resilience and later-life well-being, though causality cannot be established. The observed statistical mediation points to one possible pathway requiring longitudinal examination. Practically, should future studies confirm these associations, interventions might target both individual resilience and community walkability; culturally sensitive programs drawing on traditional beliefs could also be tested. Overall, the findings indicate directions for further research rather than prescribing specific policies or programs. Policymakers may view these ecological and cultural factors as areas meriting further investigation within ageing mental-health strategies.

## 6 Conclusion

In this cross-sectional study of community-dwelling older adults in Wenzhou, China, traditional Chinese cultural beliefs about adversity and perceived community walkability, treated as specific indicators relevant to broader cultural and environmental dimensions of resilience, respectively were positively associated with life satisfaction. Psychological resilience statistically mediated these links (accounting for 28.9 and 19.9% of the total effects, respectively), but showed no significant association with depression. Although consistent with a social-ecological view of resilience, the cross-sectional, self-report, convenience-sample design precludes causal inference; the indirect associations are best interpreted as statistical mediation only. Longitudinal and multi-region studies are needed to clarify temporal ordering and generalizability. Should future evidence support these pathways, integrated strategies addressing both individual resilience and ecological resources may warrant further investigation.

## Data Availability

The original contributions presented in the study are included in the article/supplementary material, further inquiries can be directed to the corresponding author.
